# Localization of Vehicle Back-Up Alarms by Users of Level-Dependent Hearing Protectors under Industrial Noise Conditions Generated at a Forge

**DOI:** 10.3390/ijerph16030394

**Published:** 2019-01-30

**Authors:** Rafal Mlynski, Emil Kozlowski

**Affiliations:** Department of Vibroacoustic Hazards, Central Institute for Labour Protection–National Research Institute, Czerniakowska 16, 00-701 Warsaw, Poland; emkoz@ciop.pl

**Keywords:** sound localization, level-dependent hearing protectors, back-up alarm, directivity of hearing, impulse noise, earmuffs, earplugs, auditory danger signal, warning signal, safety at work

## Abstract

The use of hearing protectors in various noisy workplaces is often necessary. For safety reasons, auditory information may be required to correctly localize the direction of an auditory danger signal. The purpose of this study was to verify if the selection of a specific level-dependent hearing protector may be important for the ability to localize a vehicle back-up alarm signal. The laboratory conditions reflected industrial conditions, under which an impulse noise was emitted against a background of continuous noise. A passive mode and a level-dependent mode (maximum and incomplete amplification) were considered. Four different models of level-dependent earmuffs and one model of level-dependent earplugs were included in the tests. The tests enabled differentiation between the individual hearing protectors. The use of earplugs in level-dependent mode did not significantly affect the ability to correctly localize the back-up alarm signal. For the earmuffs, the global assessment of the impact of a mode change revealed that, depending on the model of the earmuffs, the impact may be insignificant, but may also result in considerable impairment of the ability to localize the back-up alarm signal.

## 1. Introduction

Noise is a physical factor that affects the hearing of people who have to be in places where it is emitted. Impulse noise is a particularly dangerous type of noise due to the sudden nature of its impact [1]. This type of noise may be present during military field exercises and in industrial conditions. Under industrial conditions, impulse noise occurs most often against a background of continuous noise. Noise reduction possibilities using technical means are limited [2]. Certain activities, such as metalworking, require manual handling close to the noise source so that physically separating a person’s workplace from the noise source is impossible. The final solution, albeit the only one available, is the use of hearing protectors. Level-dependent hearing protectors are gaining in popularity and are being used more frequently. Their advantage over typical passive hearing protectors is that they do not reduce the relatively quiet sounds as much as sounds with a high sound pressure level [3]. This enables the perception of low pressure level sounds that are relevant for those using hearing protectors [4].

Level-dependent hearing protectors can be defined as a system consisting of a barrier that passively reduces the transmission of sound under these hearing protectors and from the sound transmission path in certain situations. The sound transmission path is created by means of an electronic system that reproduces the sound present in the user’s environment through a speaker placed under a hearing protector. The electronic system must be designed so that the amplification in the sound transmission path decreases as the sound pressure level of the signal present outside the hearing protector increases. The sound under the hearing protectors must be transmitted so as to ensure safe noise conditions for the user of these devices. In this respect, hearing protectors should meet the requirements set out in relevant standards, which apply to both earmuffs [5] and earplugs [6].

Some studies have been conducted on the possibility of reducing impulse noise by means of level-dependent hearing protectors [7,8,9]. It was observed that, for hearing protectors with electronic systems used in military conditions, the provision of adequate hearing protection should not be considered the only problem. Detection, recognition, identification, localization, and communication were also found to play significant sound-related roles [10]. Thus, when using hearing protectors under industrial conditions, in addition to the protective properties of hearing protectors, using the information contained in auditory danger signals is also crucially important. The presence of an electronic system used to transmit the sound under the hearing protector affects the formation of the sound reaching the users of these devices. The use of these hearing protectors may impair the ability to localize sound, i.e., recognize the direction of the source of the sound [11,12,13]. For safety reasons, under industrial conditions, it is vital to be able to correctly localize the noise source, which particularly applies to vehicle back-up alarm signals. Localization enables the user to take action to avoid being hit by a vehicle. The problem with the perception of back-up alarm signals was deemed serious enough that a dedicated electronic system for earmuffs was designed and introduced. The system functions to support the detection of such signals during the use of earmuffs in noisy environments [14]. The ability to perceive back-up alarm signals when using hearing protectors was also assessed [15]. The results of the study cited showed, with some exceptions, that the ability of normal-hearing people to locate a vehicle’s back-up alarm signal in the presence of pink noise did not improve when using level-dependent hearing protectors (one model of level-dependent earplugs and earmuffs) in comparison to using passive hearing protectors. In a different study [16], the authors checked the possibility of locating a back-up alarm signal in the presence of noise simulating quarry conditions using one specific model of hearing protector. The localization of the back-up alarm signal was found to be slightly worse when the protector was used in passive mode in comparison to when it was not used, and did not improve after the electronic system was switched on. Vaillancourt et al. [17] compared the effects of different types of signals used as a vehicle back-up alarm signal and concluded that, despite the specific advantages of a signal with broad frequency content, the use of passive hearing protectors more severely affected the detection thresholds when compared to a tonal alarm. Unfortunately, the study did not consider level-dependent hearing protectors.

The purpose of this study was to verify whether the selection of a specific level-dependent hearing protector could be important for the ability to localize the auditory danger signal, represented by a back-up alarm signal, under industrial conditions where impulse noise is generated against a background of continuous noise. We also aimed to examine the impact of changes in the amplification of the electronic system of level-dependent hearing protectors. The tests were carried out in the presence of ambient noise recorded under industrial conditions containing impulse components, which is in contrast to the previous study [11,13,14,15,17].

## 2. Materials and Methods

### 2.1. Ethics and Bioethics Commission

Prior to the commencement of this research, an application for the study was submitted to the Ethics and Bioethics Commission of the Cardinal Stefan Wyszyński University in Warsaw. The commission issued a positive review (No KEiB-22/2017) of the study, providing consent for the implementation and publication of the research results.

### 2.2. Hearing Protectors Included in the Tests

We considered commercially available level-dependent hearing protectors produced by different manufacturers including four models of earmuffs and one model of earplugs. The hearing protectors studied included: N1 (designation introduced for the purpose of this study and to retain the anonymity of the manufacturer), which were earmuffs designed for military applications, digitally controlled; N2, earmuffs designed for industrial applications, digitally controlled; N3, earmuffs designed for industrial applications, analogically controlled; N4, earmuffs designed for hunting, analogically controlled; and W, earplugs designed for industrial applications with polymer tips, digitally controlled. In the case of the earplugs, the subjects were provided two different sizes of tips. All hearing protectors carried the CE mark (means that specified European Union requirements relating to hearing protectors have been fulfilled), which is a mandatory requirement for a product to be considered as a personal protective device. The hearing protectors included in the study varied in terms of price. The most expensive was more than 11 times more expensive than the cheapest.

The tests were performed in three hearing protector operation modes: passive mode, with the level-dependent system switched off (labelled PASS); level-dependent mode, with maximum amplification in the sound transmission path (labelled LD-MAX); and level-dependent mode with the amplification set to incomplete (approximately half and labelled LD-MID).

### 2.3. Subjects

The test group consisted of 50 people. The group included an equal proportion of women and men. The age of the subjects ranged from 18 to 42 years old. The subjects qualified for the trial based on the condition of their hearing, which had to meet the requirements of EN 24869-1:1992 [18] regarding a subjective method for the measurement of sound attenuation. This standard requires that the hearing threshold should not be greater than 15 dB for frequencies up to 2000 Hz and no more than 25 dB for frequencies above 2000 Hz.

### 2.4. Back-Up Alarm

Vehicles used in Polish industrial plants are most often equipped with acoustic signaling devices to warn others about reverse driving, and they generate a tonal signal. Two types of signaling devices can be distinguished that differ in the location of the dominant spectral components of the signal generated. These can be in the 1/3-octave band with a center frequency of 1250 Hz or 3150 Hz. The dominant spectral components in the first type are within the range of 500 to 1500 Hz, as specified in ISO 7731:2003 [19], in reference to one of the conditions to be met by an auditory danger signal. In the tests, we used a vehicle’s back-up alarm signal that met the required standards.

**Figure 1:**
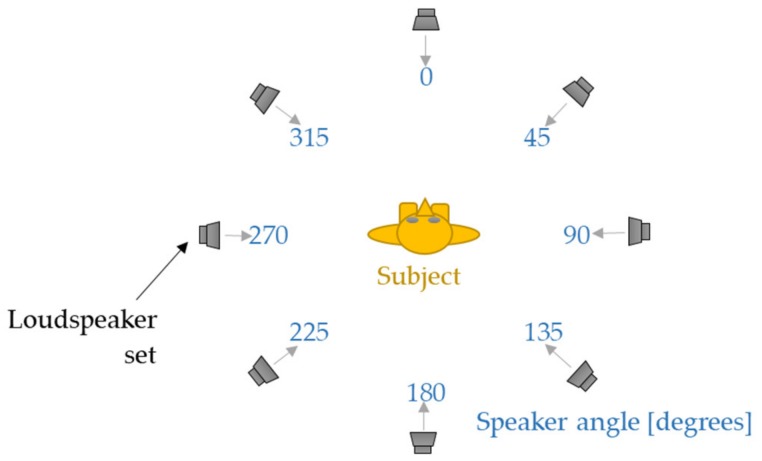
The directions from which the back-up alarm signal was reproduced and the location of the subject in the experimental setup.

The system designed to reproduce the back-up alarm signal was based on eight M-Audio Bx5 D2 loudspeaker sets (inMusic Brands, Cumberland, RI, USA) that were placed at the height of a sitting person’s head. The M-Audio loudspeaker sets were evenly distributed in eight directions, every 45°, where the first loudspeaker set was placed directly in front of the subject’s face. This direction was marked as 0°. Figure 1 provides a graphic representation of the directions from which the back-up alarm signal was reproduced. The distance between the loudspeaker sets and the point determined by the center of the person’s head was 1.8 m. The electrical signal was fed to the inputs of the M-Audio loudspeaker sets from a MOTU 24I/O audio interface (MOTU, Cambridge, MA, USA). The sampling frequency was 44,100 Hz. For the purpose of testing the perception of back-up alarm signals, 15 different sequences of these signals were prepared. The order of signal reproduction from individual directions was randomly determined. The test of each elementary measurement situation was performed based on a sequence of 24 instances of a back-up alarm signal, as this signal was reproduced from eight directions, and the measurement was repeated three times in each of the directions.

### 2.5. Acquisition of the Direction Indications Provided by Subjects

The subjects indicated the directions from which a back-up alarm signal was received by pressing a button on a panel of eight push buttons arranged in a circle, reflecting the possible locations of the sound source. The response panel has been described in detail in a previous study [20].

### 2.6. Reflecting Industrial Noise Conditions

To reflect the presence of noise generated in industrial situations under laboratory conditions, a virtual acoustic environment using ambisonic technology was developed. The experimental setup was located in an acoustic chamber in the Tech-Safe-Bio CIOP-PIB Laboratory (Central Institute for Labour Protection—National Research Institute, Warsaw, Poland) [21]. The ambient noise obtained was reproduced in the experimental setup based on the recording of noise generated during metal processing at a forge. The ambient noise consisted of acoustic impulses generated by drop-forging hammers against a background of continuous noise. The recording was performed with the use of a Sennheiser AMBEO VR Mic ambisonic microphone (Sennheiser Electronic GmbH & CO KG, Wennebostel, Germany) connected to a Tascam DR-680 MkII recorder (TEAC Corporation, Tokyo, Japan).

**Figure 2:**
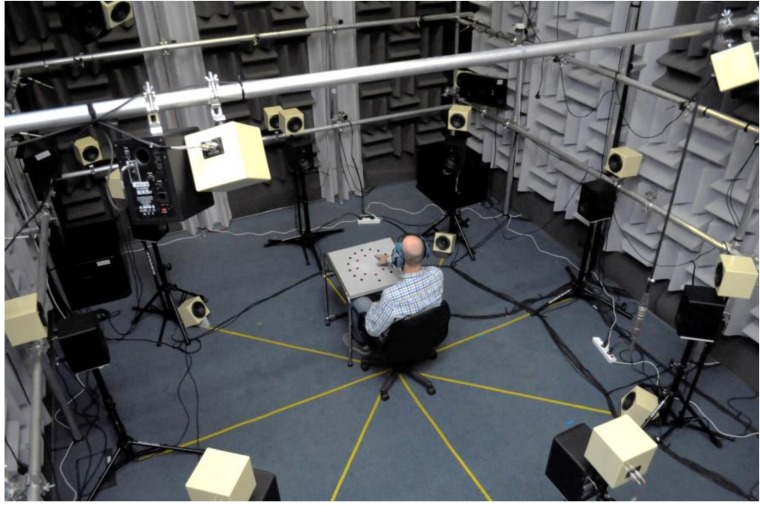
The experimental setup during the back-up alarm signal localization tests, performed with the participation of a subject wearing a level-dependent hearing protector.

A total of 17 Avantone MixCube loudspeakers (Avantone Pro, Tallman, NY, USA) were placed in the test room to reproduce ambient noise. The loudspeakers were located in a sphere with a 2-m radius in relation to the center of the seated person’s (the subject’s) head. Eight loudspeakers were placed circumferentially directly above the M-Audio BX5 D2 loudspeaker sets (inMusic Brands). Another four Avantone MixCube loudspeakers (Avantone Pro) were placed on the floor, and another four above the test subject (45 degrees upward in relation to the level of the subject’s head). The 17th loudspeaker was placed directly above the subject’s head. The sound reproduction system was supplemented with two sets of Nexo LS600/PS8 subwoofers (Nexo, Plailly, France). A photograph of the experimental setup during the tests is shown in Figure 2. The ambient noise was reproduced using a Rapture 3D Ambisonic Player (Blue Ripple Sound Limited, London, UK) at a sampling frequency of 48 kHz.

The A-weighted equivalent sound pressure level of the ambient noise reproduced on the experimental setup was 84.8 dB. The C-weighted peak sound pressure level was 111.8 dB. The values of the noise parameters were monitored before the beginning of each measurement session. The measurements were recorded at the location of the subject’s head while the subject was absent by using a SVAN 979 Class 1 sound level meter (SVANTEK Sp. z o.o., Warsaw, Poland).

### 2.7. Test Method

Before commencing the tests, each subject completed a training session. Each subject participated in the measurements for all five hearing protectors (listed in Section 2.2) in each of the three operation modes.

Industrial noise conditions were reflected during the tests. The ambient noise was reproduced according to the rules defined in Section 2.6. A back-up alarm signal was reproduced against the background of this noise according to randomly defined sequences (as described in Section 2.4). The task of the subject sitting in the center of the experimental setup was to indicate the direction of the back-up alarm signal by pressing the appropriate key on the panel (as described in Section 2.5). During the tests, each subject provided 360 indications (a sequence of 24 signals in each basic measurement situation for the three operation modes of each of the hearing protectors, for five different hearing protectors).

To analyze the obtained data, we created a direction recognition index for the back-up alarm signal. This index expresses, as a percentage, the ratio of the number of correct indications of the direction (or directions) in a given measurement situation to the number of all reproductions of the back-up alarm signal from the direction (or directions) considered in this situation.

### 2.8. Statistical Analysis

To determine which changes in the value of the direction recognition index between particular measurement situations should be considered significant, we completed a statistical analysis of the obtained data. For this purpose, a Wilcoxon test (equivalent to the Mann-Whitney *U* test) was used. The calculations were performed using MATLAB R2017b (version 9.3) with the Statistics and Machine Learning Toolbox (MathWorks Inc., Natick, MA, USA).

## 3. Results

### 3.1. Distribution of Indications between Individual Directions

**Figure 3:**
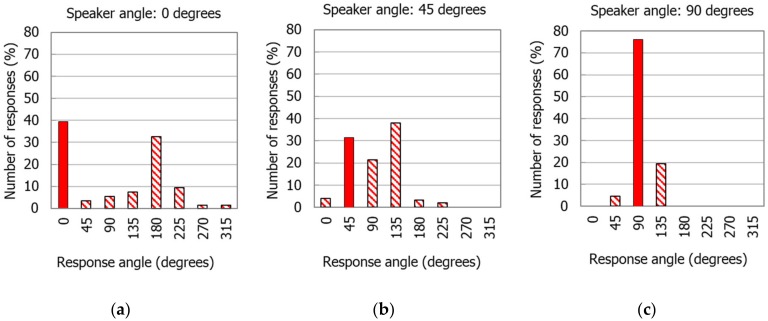
Distribution of the indications given by users of the N2 earmuffs in the LD-MAX mode when the back-up alarm signal was reproduced from a certain angle: (**a**) 0°, (**b**) 45°, and (**c**) 90°. LD-MAX—level-dependent mode, with maximum amplification in the sound transmission path.

The first step of the back-up alarm signal perception assessment involved analyzing how the indications of the subjects changed if the signal was reproduced from a specific direction. This analysis enabled the identification of the regularity of level-dependent earmuffs (N1–N4). In situations where the danger signal was reproduced from the 0° and 180° directions, in the majority of cases, a significant number of indications did not only reflect the direction from which the signal was played. Correct indications of the 0° direction were noted for 20–40%, 31–47%, and 25–45% of cases for earmuffs used in the PASS, LD-MAX, and LD-MID modes, respectively. Correct indications of the 180° direction were slightly more frequent and their numbers expressed as a percentage were 45–53%, 26–53%, and 26–67% for the PASS, LD-MAX, and LD-MID modes, respectively. In the case of these two back-up alarm signal reproduction directions, 0° and 180°, the most frequent errors were of the ‘front–back’ type. This means that the subjects had problems in determining whether the back-up alarm signal came from in front or from behind. This situation is represented by the example in Figure 3a, where, for the back-up alarm signal from the 0° direction, the indications of this direction given by all subjects accounted for 39% of all indications, whereas the indications of the 180° direction were only six percentage points lower, representing 33% of all indications. Indications for the other directions in any situation did not exceed 10% of the overall number of indications.

An even worse situation occurred when the back-up alarm signal was reproduced from the directions at the angles of 45° and 315°. In these directions, the direction of the signal was reproduced was not the one that was indicated the most often. The subjects made errors in changing the direction from the front to the direction from the back of the person and indicated the directions of sound as adjacent to the directions from which the signal was played. An example of this situation is shown in Figure 3b, where, in the case of the back-up alarm signal coming from the 45° direction, the correct indications of this direction identified by all subjects were not the most frequent (31%). A larger number of indications, at 38%, was in the direction deviated from the axis crossing the person’s ears by 45°; however, not from the front, but from beyond at 135°. Therefore, these were front–back type errors. A significant proportion of indications (21%) were in the direction adjacent (90°) to the direction from which the signal to be recognized was reproduced.

However, in the case of directions located to the side of the person, i.e., from directions at angles of 90° and 270°, the situation was different, since the majority of the indications of the subjects when the signals were reproduced from these directions were correct. Therefore, the correct indication of the 90° direction occurred in up to 81% of cases. This occurred when using the N3 earmuffs in LD-MAX mode. This was also the case for the 270° direction. In the case of these two directions (90° and 270°), the histograms were the slimmest. An example of this is shown in Figure 3c, where, in the case of the back-up alarm signal from the direction at the 90° angle (left side of the person), the correct indications of this direction given by all subjects were by far the most frequent, representing 76% of the total. For the other two directions of 45° and 135° (adjacent to direction 90°), 5% and 19% of indications were given, respectively.

**Figure 4:**
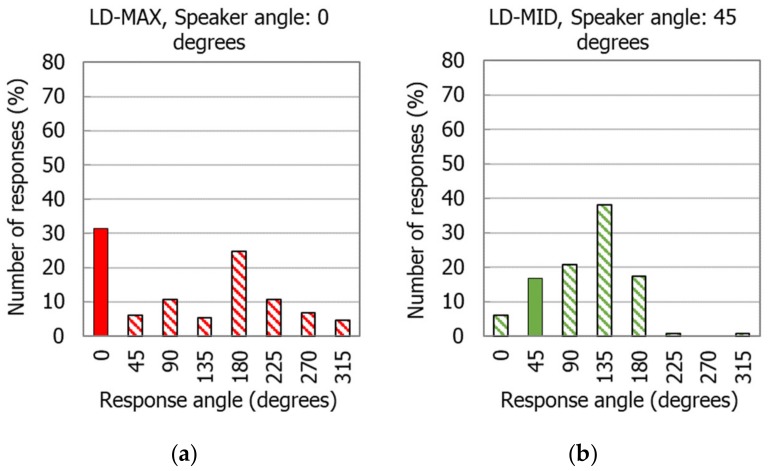
Distribution of the indications given by users of the N4 earmuffs, when the back-up alarm signal was reproduced from a certain angle: (**a**) 0° (earmuffs used in the LD-MAX mode) and (**b**) 45° (earmuffs used in the LD-MID mode). LD-MAX—level-dependent mode, with maximum amplification in the sound transmission path; LD-MID—level-dependent mode with the amplification set to incomplete (approximately half).

The widest histogram distributions were obtained with the N4 earmuffs and indications regarding the reproduction of the back-up alarm signal from the directions at angles 0° and 180°. In such situations, the indications ranged over all eight directions. An example of this situation is shown in Figure 4a. For the earmuffs (N4), there were a significant number of front–back errors, which occurred for the back-up alarm signal reproduction directions at the angles of 45° and 315°. Then, in all operation modes of the N4 earmuffs, the majority of subjects responded that the signal came from the direction of 135° instead of 45° (as shown in Figure 4b) and similarly from the direction of 225° instead of 315°.

In the case of the W earplugs, the situation was different from the earmuffs. The direction most frequently indicated by the subject was almost always the direction from which the back-up alarm signal was reproduced. The histogram bar showing the direction from which the signal was reproduced was almost always (23 out of 24 histograms) the highest of the eight directions considered. For the directions from angles of 90° and 270° (at the side of the person), nearly 90% of the indications were correct, whereas the lowest frequency of correct direction recognition (37%) was for the angle of 0° when the earplugs were used in passive mode.

**Figure 5:**
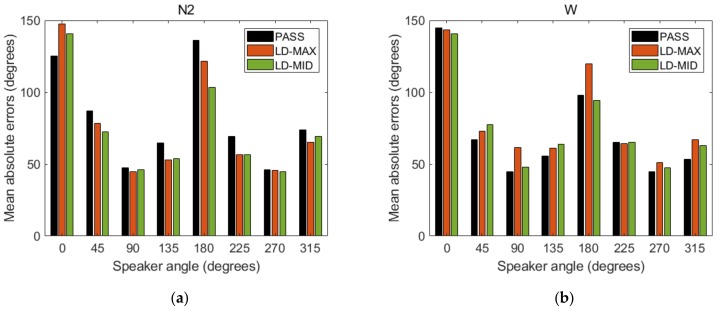
Mean absolute errors calculated for the indications given by users of the N2 earmuffs (**a**); and W earplugs (**b**). PASS—with the level-dependent system switched off. LD-MAX—level-dependent mode, with maximum amplification in the sound transmission path; LD-MID—level-dependent mode with the amplification set to incomplete (approximately half).

Similar direction recognition observations could be made when the signal came from the front/behind or from side of the person were also made in mean absolute error analysis. Mean absolute error was calculated as the mean angular distance between response and the direction from which signal was reproduced. The examples of error values are shown in Figure 5a,b, respectively, for N2 earmuffs and W earplugs. The greatest errors were observed for angles of 0° and 180°, regardless of the model of the level-dependent hearing protector. For angles of 90° and 270° mean error values were the smallest and they were approximately equal to the angular resolution used in the experiment (45°).

### 3.2. Direction Recognition Index

#### 3.2.1. Global Index Values

During the tests, all subjects provided a total of 18,000 indications; 8181 of which were correct, meaning the directions of the back-up alarm signal were correctly identified. Therefore, the global direction recognition index of the back-up alarm (as defined in Section 2.7) was 45.5%. For each of the hearing protectors, the subjects provided 3600 indications. The values of the direction recognition index determined for all results obtained for each hearing protector were: 41.6% (N1), 45.0% (N2), 45.9% (N3), 38.6% (N4), and 56.1% (W). The numbers indicate that the correct recognition of the direction of the back-up alarm signal is possible in more cases when level-dependent earplugs are used rather than level-dependent earmuffs.

#### 3.2.2. Index Values Broken Down by the Different Modes of Using Hearing Protectors

**Figure 6:**
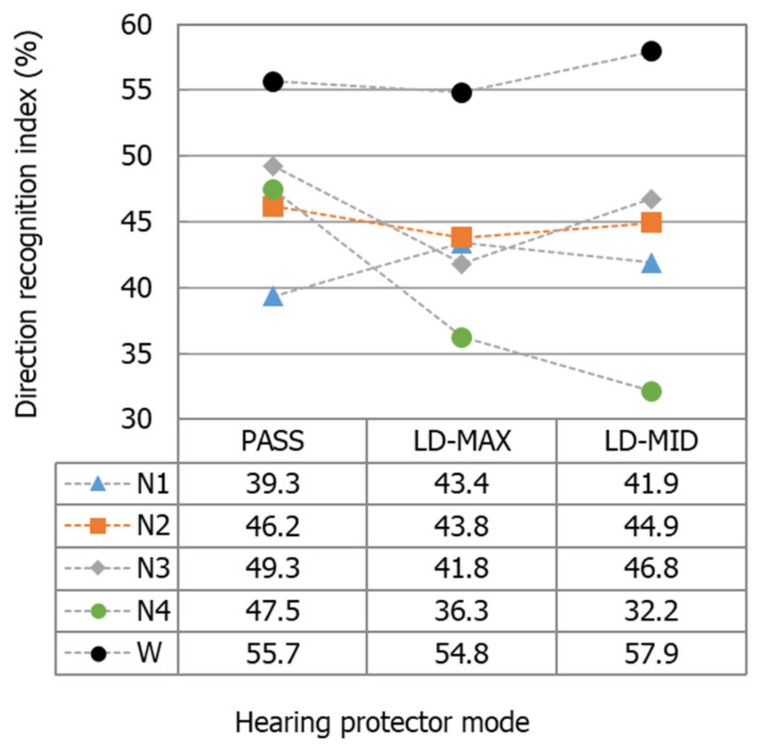
Values of the direction recognition index determined for all of the measurement data obtained for each of the hearing protectors with a breakdown by the operation mode of these protectors. PASS—with the level-dependent system switched off. LD-MAX—level-dependent mode, with maximum amplification in the sound transmission path; LD-MID—level-dependent mode with the amplification set to incomplete (approximately half).

To compare the influence of different modes of level-dependent hearing protectors on the ability to correctly recognize the direction of sound, the values of the direction recognition index were determined with a breakdown by these modes. The values obtained are shown in Figure 6. Every point in the chart was created based on the 1200 indications provided by the subjects. Overall, the use of earplugs enabled the correct localization of the back-up alarm signal in a greater number of cases than with earmuffs, regardless of the operation mode of the hearing protectors. Differences in the number of correct indications of the back-up alarm signal direction between the different operation modes of the hearing protectors was as low as 2.3 percentage points (N2 earmuffs). However, these differences could exceed 15 percentage points (N4 earmuffs).

#### 3.2.3. Values of the Index Determined by Breakdown by Different Directions of Back-Up Alarm Signal Reproduction

**Figure 7:**
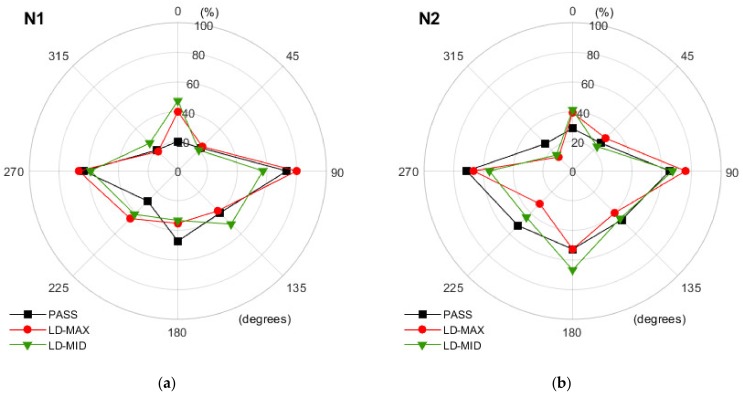
The values of the direction recognition index determined for different directions when using earmuffs in different modes: (**a**) N1 and (**b**) N2. PASS—with the level-dependent system switched off. LD-MAX—level-dependent mode, with maximum amplification in the sound transmission path; LD-MID—level-dependent mode with the amplification set to incomplete (approximately half).

**Figure 8:**
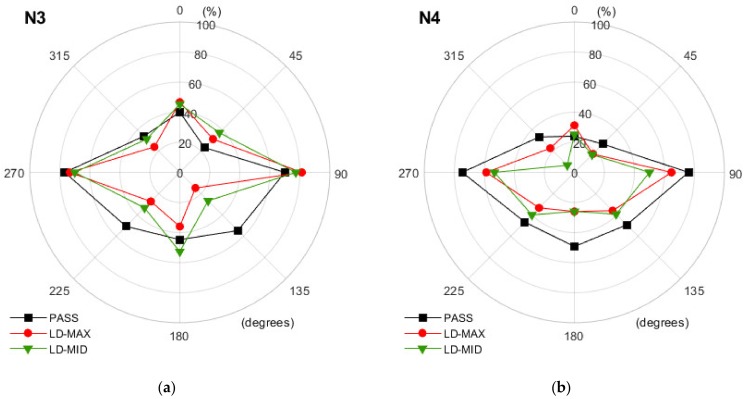
The values of the direction recognition index determined for different directions when using earmuffs in different modes: (**a**) N3 and (**b**) N4. PASS—with the level-dependent system switched off. LD-MAX—level-dependent mode, with maximum amplification in the sound transmission path; LD-MID—level-dependent mode with the amplification set to incomplete (approximately half).

**Figure 9:**
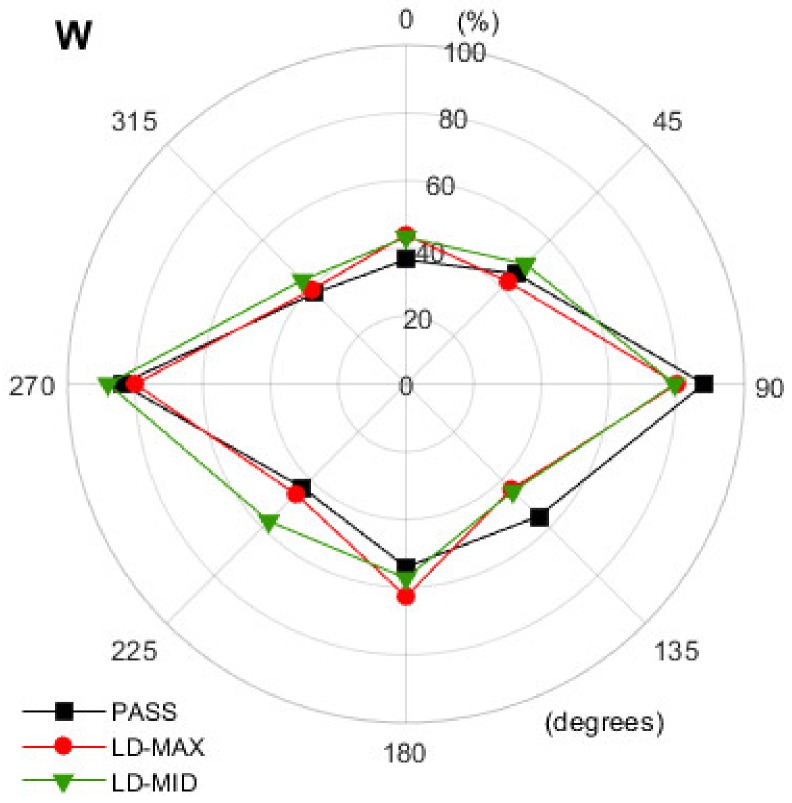
The values of the direction recognition index determined for different directions when using the W earplugs in different modes. PASS—with the level-dependent system switched off. LD-MAX—level-dependent mode, with maximum amplification in the sound transmission path; LD-MID—level-dependent mode with the amplification set to incomplete (approximately half).

The diagrams shown in Figure 7, Figure 8 and Figure 9 present the results obtained in each of the three operation modes of the hearing protectors with a breakdown by the individual directions of the back-up alarm signal source. The results of the analysis, indicating which changes in the values of the direction recognition index should be deemed as significant, are presented in Table 1.

Analyzing the data in Figure 7, Figure 8 and Figure 9, we confirmed that users of level-dependent hearing protectors have the least difficulty in correctly indicating the direction of the back-up alarm signal when the signal is reproduced from the side of the person (angles 90° and 270°). The values of the direction recognition index were lower when the sound was played behind a person and the lowest when it came from directly in front of a person.

When using level-dependent hearing protectors, it is important to determine whether the change in the operation mode of the protector significantly affects the user’s ability to correctly localize the back-up alarm signal. The data presented in Figure 7, Figure 8 and Figure 9 indicate that, in many situations, a change in the mode of use of a hearing protector does not affect the direction recognition index. However, this is not a rule. For example, in the case of the N1 earmuffs (Figure 7a) and the 0° direction, the change from passive (PASS) to level-dependent mode led to a 27 percentage points and 20 percentage points increase in the number of correct indications for LD-MID and LD-MAX, respectively. These increases in the direction recognition index values, according to the data from Table 1, were statistically significant. Whereas, in the case of 90°, the change from the PASS to LD-MID mode resulted in a reduction in the number of correct indications by 16 percentage points, while at the angle of 180°, both variants of using the N1 earmuffs in level-dependent mode were less advantageous than in passive mode. This case was in contrast to the one observed for the angle of 225°. Here, in contrast to passive mode, both level-dependent modes were advantageous. The increase (statistically significant) was 13 percentage points (LD-MID) and 17 percentage points (LD-MAX). The results showed that the use of level-dependent mode relative to passive mode, depending on the specific angle at which the back-up alarm signal was received, resulted in an increase or decrease in the number of correct indications of the direction. In the case of the N1 earmuffs, statistically significant changes occurred in 7 out of 16 possible cases (eight related to the LD-MAX mode and eight to the LD-MID mode). An increase in the number of correct indications was observed in four cases and a decrease in three. A similar situation occurred in the case of the N2 earmuffs (Figure 7b), where statistically significant changes occurred in 7 out of 16 cases. In three of these cases, the number of correct indications increased, and in four cases, this number decreased. The use of the N3 earmuffs resulted in only 6 of 16 statistically significant increases (two cases) or decreases (four cases) in the number of correct indications. With the N3 earmuffs (Figure 8a), the reduction in the direction recognition index related to the operation in level-dependent mode in contrast to passive mode produced relatively high values ranging from 17 to 40 percentage points, observed for the angles of 135° and 225°. The values of the advantageous changes (increase in the value of the direction recognition index) were 14 (LD-MID, 45°) and 11 (LD-MAX, 90°) percentage points. For the N4 earmuffs (Figure 8b), a significant number of cases, 11 out of 16 possible, were identified where the impact of changing the earmuff’s mode from the PASS to the LD-MAX or LD-MID was statically significant. All 11 changes were unfavorable; after activating the level-dependent mode, we observed that the number of correct indications of the back-up alarm signal direction decreased.

Results obtained during the use of W earplugs (Figure 9) indicate that a change in the mode of their use was of little significance. Statistically significant changes in the direction recognition index occurred only after the change from the PASS to LD-MID mode at the angles of 90° (decrease by 9 percentage points) and 225° (increase by 14 percentage points).

## 4. Discussion

The analysis of the changes in the indications of the 50 subjects between the individual eight directions shows that, for the 0° angle (straight in front of the person) and 180° (behind the person), in the majority of cases using level-dependent earmuffs, a considerable number of ‘front–back’ errors were observed. The people who participated in the study had problems in determining whether the back-up alarm signal came from the front or was possibly from behind. These types of errors (in addition to the indication of adjacent directions) were also frequent in the case of the directions of 45° and 315°. These situations were opposite to the cases when the back-up alarm signal was played from directions located at the sides of a person, i.e., from directions at the angles of 90° and 270°. The direction of signals reproduced from these directions was indicated correctly in the vast majority of cases.

These results provide the first significant differentiation between level-dependent earmuffs and earplugs. For the earplugs, in nearly all of the situations considered, the direction from which the signal was reproduced was indicated almost always more frequently than the remaining seven directions. For the earmuffs, this was not always true because, for the angles of 45° and 315°, the direction from which the signal to be recognized was reproduced was not indicated most frequently. The conclusions resulting from the distribution of indications for the individual directions are partly consistent with other studies, where the authors claimed that the problems in locating the sources of sound occurred particularly often on the front–back axis [22]. In addition, the back direction is indicated by the subjects less frequently when the sound comes from the front rather than the other way around [23]. In this study, the correct indications of the 180° direction were insignificantly more frequent than for the 0° direction. However, this pertained to different situations than the work mentioned above, i.e., during the use of hearing protectors.

Little data exist on sound direction recognition tests for level-dependent hearing protectors. However, the results obtained in this study can be referred, to a limited extent, to the data available. A study where the direction of sound reproduction of cocking an AK-47 (70 dB) in the presence of broadband masking noise (55 dB) was analyzed, presented the indications of 10 subjects with the reproduction of the test signal from directions 30° apart [13]. The analysis of the diagrams regarding the use of level-dependent earplugs (EB-15 earplugs and earplugs designed by the authors of the above-mentioned study) enabled the identification of a similar distribution of direction indications to the ones obtained in this paper. Despite of different test conditions, for the direction at the angle of 0°, a significant number of indications fell not only in this direction, but also at 180°. The same was true when the sound was reproduced from the direction of 180°. In addition, for the 30° direction, Brown et al. [13] reported the occurrence of front–back errors and the indication of adjacent directions, which is similar to the situation that occurred in this study when the back-up alarm signal was reproduced from the 45° direction. We observed that the relatively smallest diversity of indications in Brown et al. [13], where the test signal was reproduced from directions of 90° and 270°, corresponded to the slimmest shapes of the graphs in the histograms obtained in this study (Figure 3c).

In this study, we determined the direction recognition index for the back-up alarm signal. The values of this index, which were determined with a breakdown by hearing protector operation modes (i.e., set globally without dividing the individual directions of the alarm signal), showed that regardless of the mode of these protectors, it is possible to correctly localize the back-up alarm signal in a much larger number of cases when level-dependent earplugs are used compared to when level-dependent earmuffs are used. The average value of the index for the earplugs (with consideration of all three modes of operation) exceeded the average value for all four earmuffs and their modes of operation by more than 13 percentage points. The range of the direction recognition index values between the different operation modes of the hearing protectors was relatively small (two to four percentage points) for the N1 and N2 earmuffs and the W earplugs. Slightly greater differences in the direction recognition index between the modes were observed for the N3 earmuffs (seven percentage points). The largest span, exceeding 15 percentage points, was observed for the N4 earmuffs.

This global analysis of the results indicated that among the hearing protectors, the operation mode for some (N1, N2 earmuffs and W earplug) has an insignificant impact on the user’s ability to localize the back-up alarm signal. The mode of use of the hearing protectors may also influence, to a certain extent, the ability to recognize the direction of a sound. This was the case with the N3 earmuffs, for which the level-dependent mode resulted in a noticeable deterioration in the ability to localize the back-up alarm signal. Additionally, the type of hearing protectors may significantly influence the possibility of recognizing the direction of back-up alarm signals. Hearing protectors may include those (N4) that significantly impair the user’s ability to recognize the direction of the sound when switched from passive to level-dependent mode. This deterioration occurred with both the maximum and incomplete amplification in the level-dependent system of the N4 earmuffs, and amounted to above 11 and 15 percentage points in comparison to passive mode, respectively.

The results published in Alali and Casali [15] can be compared with the values of the direction recognition index measured as part of this study. In Alali and Casali [15], the correct recognition of the direction of the signal in the presence of pink noise with an A-weighted sound pressure level of 90 dB was 47.7% for the level-dependent earmuffs and 62.2% for the level-dependent earplugs. Within the framework of this study, for earmuffs, the direction recognition index (global values) ranged from 38.6% to 45.9%, and 56.1% for the earplugs. Considering the differences in the test conditions and the test facilities themselves, the results obtained can be regarded as comparable. In another study [11] where the recognition of sound directions, including the front–back, left-right, and up-down directions, was examined, the results from 20 subjects showed that the number of correct indications for level-dependent earplugs was about 42.5% and 31.5% for level-dependent earmuffs. Again, the test conditions (eight loudspeakers that were the source of the test signal with a 230 ms burst of wideband noise) or the models of the hearing protectors differed from those in this study, but the tendency of the relationship between the earplugs and the earmuffs is similar.

The determination of the direction recognition index for the back-up alarm signal, broken down by direction, enabled repeating the previous general observation that users of level-dependent hearing protectors have the least difficulty in correctly indicating the direction of the back-up alarm signal when it is reproduced from the side of a person (angles of 90° and 270°). The values of the direction recognition index were lower when the sound was played behind a person (180°) and the lowest when the source was directly in front of the person (0°). For the number of errors falling on the directions of 0° and 180°, and 90° and 270°, a similar distribution of results was obtained in the aforementioned study [13]. The root mean square error (RMSE), defining the mean angular distance of responses from the target angle, for the level-dependent EB-15 earplugs was about 38° and 55°, when the sound came from the front and back directions, respectively, and about 9° and 11° when the sound came from the right and left sides of a person, respectively. However, these results are not fully comparable with the results of our study as a different measure was applied to different models of test earplugs and different test conditions. Nevertheless, the ratio of the average number of incorrect indications of the front–back directions to incorrect indications of the left–right directions in both studies indicated the same trend, which was 3.1 in this study and 4.7 for the cited study [13].

Alali and Casali [15] demonstrated, with some exceptions, that the ability of normal-hearing people to locate a vehicle’s back-up alarm signal in the presence of pink noise did not improve when using level-dependent hearing protectors (earplugs and earmuffs) in comparison to the use of passive hearing protectors. A similar finding was reported by Giguère et al. [16], where the situation did not improve after switching from passive mode to the electronic system mode. This conclusion was also reached in this study with regard to earplugs, where their operation mode had practically no impact on the measured values of the direction recognition index. For earmuffs, in this study and in certain cases, the change from passive mode to level-dependent mode (maximum or incomplete amplification in the level-dependent system) resulted in statically significant differences in the value of the direction recognition index. During the use of the N1 and N2 earmuffs, statistically significant changes occurred in 44% of cases; however, the number of changes resulting in increasing the direction recognition index value roughly balanced the number of changes resulting in a decrease in the index value. Using the N3 earmuffs in level-dependent mode resulted in a change in the direction recognition index in almost 38% of the cases, whereas the deterioration of the ability to recognize the direction was twice more often (four instances of decreases) than the improvement (two instances of increases) when compared to passive mode. A slightly different situation was observed for the N4 earmuffs, where there was a total of 69% of cases where the ability to correctly recognize the direction of sound was statistically significantly different from passive mode, but all of the changes were negative, meaning the changes resulted in a decrease in the value of the direction recognition index. The analysis of the use of earmuffs in level-dependent mode in comparison to passive mode, with a breakdown by different directions, demonstrated that in a certain number of cases (from 38% to 69%), the mode affected the ability of the users to localize the back-up alarm signal, which differed from the conclusion drawn in the cited study [15]. Notably, the authors [15] used different ambient noise (pink noise with A-weighted sound pressure levels of 60 dB and 90 dB), a different back-up alarm signal, and included hearing protectors (one model of a specific type of hearing protector) other than the ones used in this study.

This issue is important from the perspective of ensuring safety in a workplace. The methodology used in this study can be used to assess the ability of workers using level-dependent hearing protectors to recognize the direction of an auditory danger signal. Test results indicate that the model of a level-dependent hearing protector available to a worker will significantly affect their ability to localize the vehicle’s back-up alarm signal. However, the tests carried out under laboratory conditions cannot fully replicate the conditions encountered in an industrial facility. The localization of auditory danger signals by users of level-dependent hearing protectors may be, to some degree, worse in real-life conditions than in experiments conducted in a laboratory because the attention of the subjects who participated in the experiment was entirely focused on the task of sound localization. In real industrial conditions, however, the workers are focused on their jobs.

## 5. Conclusions

The tests enabled the differentiation of the level-dependent hearing protectors in terms of the ability to assess the direction of an auditory danger signal, represented by a back-up alarm signal, by the user of these protectors under industrial conditions where impulse noise is generated against a background of continuous noise.

We found that the operation mode of level-dependent earplugs, i.e., passive or level-dependent, did not significantly affect the ability to correctly indicate the direction of a back-up alarm signal.

The assessment of the earmuffs was complicated. The analysis of a breakdown by the individual directions indicated that, in a significant number of cases, there was a change in the operation mode result depending on the direction of the back-up alarm signal, either by an increase or decrease in the ability to properly recognize this direction. The global assessment showed that depending on the model of the earmuffs, the impact of switching on level-dependent mode may be insignificant, but it may be clearly noticeable, or even result in a significant deterioration of the ability to recognize the direction of the back-up alarm signal.

The above conclusions on level-dependent earplugs and earmuffs mean that, in workplaces where it is important for the safety of a worker using hearing protection to correctly recognize the direction of a back-up alarm signal, the choice of a specific model of these protectors is of crucial importance.

## Figures and Tables

**Table 1:** Determined *p*-values for comparisons between different modes of using level-dependent hearing protectors for individual angles. PASS—with the level-dependent system switched off. LD-MAX—level-dependent mode, with maximum amplification in the sound transmission path; LD-MID—level-dependent mode with the amplification set to incomplete (approximately half).

Angle (°)	PASS–LD-MAX	PASS–LD-MID	LD-MAX–LD-MID	PASS–LD-MAX	PASS–LD-MID	LD-MAX–LD-MID
		**N1** ^1^			**N2**	
0	**<0.01** ^2^	**<0.01**	0.20	0.05	**0.02**	0.73
45	0.78	0.67	0.48	0.37	0.51	0.12
90	0.17	**<0.01**	**<0.01**	**0.04**	0.72	0.10
135	0.72	0.06	**0.03**	0.25	0.82	0.35
180	**0.04**	**0.01**	0.72	1.00	**0.01**	**0.01**
225	**<0.01**	**0.02**	0.49	**<0.01**	0.17	**0.02**
270	0.55	0.41	0.15	0.38	**0.01**	0.06
315	0.77	0.17	0.10	**0.01**	**0.02**	0.62
		**N3**			**N4**	
0	0.25	0.35	0.82	0.16	0.79	0.25
45	0.12	**0.01**	0.28	0.05	**0.04**	0.88
90	**0.02**	0.15	0.39	**0.03**	**<0.01**	**0.01**
135	**<0.01**	**<0.01**	**0.01**	**0.02**	0.08	0.55
180	0.13	0.17	**<0.01**	**<0.01**	**<0.01**	1.00
225	**<0.01**	**<0.01**	0.26	**0.02**	0.25	0.23
270	0.51	0.19	0.52	**<0.01**	**<0.01**	0.35
315	0.06	0.62	0.16	**0.04**	**<0.01**	**<0.01**
		**W**				
0	0.20	0.24	0.91			
45	0.56	0.49	0.20			
90	0.06	**0.04**	0.89			
135	0.05	0.07	0.91			
180	0.13	0.56	0.35			
225	0.64	**0.02**	0.05			
270	0.37	0.32	0.06			
315	0.81	0.35	0.48			

^1^ Hearing protector; ^2^ *p*-Values for comparisons that are significant (*p* < 0.05) are in bold font.
